# Focusing on Formononetin: Recent Perspectives for its Neuroprotective Potentials

**DOI:** 10.3389/fphar.2022.905898

**Published:** 2022-05-30

**Authors:** Jiao Tian, Xing-Qin Wang, Zhen Tian

**Affiliations:** ^1^ Department of Infection, Ministry of Education Key Laboratory of Child Development and Disorders, National Clinical Research Center for Child Health and Disorders, China International Science and Technology Cooperation Base of Child Development and Critical Disorders, Children’s Hospital of Chongqing Medical University, Chongqing, China; ^2^ Chongqing Key Laboratory of Translational Medical Research in Cognitive Development and Learning and Memory Disorders, Chongqing Key Laboratory of Child Infection and Immunity, Children’s Hospital of Chongqing Medical University, Chongqing, China; ^3^ Department of Neurosurgery, Nanfang Hospital, Southern Medical University, Guangzhou, China; ^4^ Department of Pharmacology, College of Pharmaceutical Sciences, Southwest University, Chongqing, China; ^5^ Department of Pharmacy, Tangdu Hospital, Fourth Military Medical University, Xi’an, China

**Keywords:** neurological disorders, formononetin, Alzheimer’s disease, dementia, traumatic brain injury, nociception, neuroinflammation, oxidative stress

## Abstract

Nervous system is the most complex system of the human body, hence, the neurological diseases often lack effective treatment strategies. Natural products have the potential to yield unique molecules and produce integrative and synergic effects compared to standard therapy. Mounting evidence has shown that isoflavonoids contained in traditional medicinal plant or dietary supplementation may play a crucial role in the prevention and treatment of neurological diseases due to their pronounced biological activities correlating to nervous system. Formononetin, a non-steroidal isoflavonoid, is a bioactive constituent of numerous medicinal plants such as red clover (*Trifolium pratense*) and *Astragalus membranaceus.* Emerging evidence has shown that formononetin possesses considerable anti-inflammatory, antioxidant and anti-cancer effects. This review intends to analyze the neuropharmacological potential of formononetin on the therapy of nervous system disorders. The neuroprotective properties of formononetin are observed in multiple neurological disorders including Alzheimer’s disease, dementia, cerebral ischemia, traumatic brain injury, anxiety, and depression. The beneficial effects of formononetin are achieved partially through attenuating neuroinflammation and oxidative stress *via* the related signaling pathway. Despite its evident effects in numerous preclinical studies, the definite role of formononetin on humans is still less known. More well-designed clinical trials are required to further confirm the neuroprotective efficacy and safety profile of formononetin before its application in clinic.

## Introduction

Nervous system is the most complex system of the body with quite a few functions still unknown. Neurological diseases are the pathological states that affect either the peripheral nervous system, spinal cord or brain and finally cause functional disorders (Gunata et al., 2020). The etiology is complicated, numerous factors including genetics, trauma, infection, tumor, immunological factors can all lead to the dysfunction of nervous system and cause neurological disorders such as neuropsychiatric disease, neurodevelopment disease and neurodegenerative disease (Upadhyay, 2014). Routine therapies are generally not potent enough in preventing the progression of neuronal diseases, and the cure is even more difficult. Therefore, it is necessary to develop alternative medicaments for treating these diseases.

As an important part of medicine, herbs have been used to treat various diseases for centuries in some Asian countries, especially China and India (Sharma et al., 2018; Wang et al., 2022). However, because of the unclear components and mechanisms, traditional phytomedicine has not been fully admitted and accepted in many countries. With the development of modern medicine, the mysteries surrounding herbs are gradually being uncovered. More and more attention is being focus on the application of herbal medicine in treating complicated diseases because of their distinctive efficacy (Rastogi et al., 2016; Liu et al., 2022). Multiple plants and herbs in traditional Chinese and India Ayurvedic systems (e.g., *Panax notoginseng*, *Shankhapushpi*, *Guggulu*, *Yashtimadhu*, *Centella asiatica*) have been shown to be beneficial for controlling the symptoms and related disorders of neurological diseases (Sharma et al., 2019). Compared with the synthetic drugs, which cannot control the gradual progression of neurological diseases in most cases, herbs may have the potential to eliminate the root cause of diseases rather than just alleviate the symptoms because of their synergic and integrative effects. In addition, synthetic drugs often have undesirable but inevitable adverse effects, affecting patients’ compliance and treatment outcome. However, there are no similar concerns about the phytomedicine owing to their natural source and relatively safety profile (Sharma et al., 2018). The comprehensive overview about the beneficial roles of medicinal herbs over synthetic drugs in neurological disorders can refer to these excellent reviews (Sharma et al., 2018; Sharma et al., 2019; Sharma et al., 2021).

**FIGURE 1:**
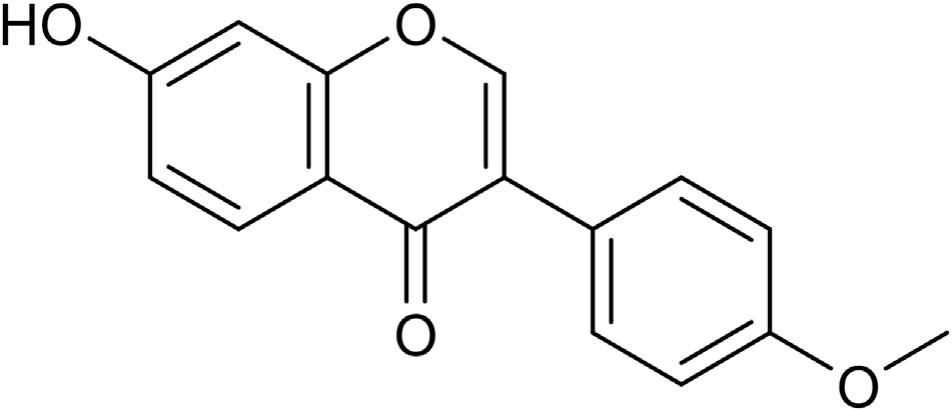
The chemical structure of formononetin.

Red clovers *Trifolium pratense L.* is a common perennial herb that has traditionally been used to treat some skin and respiratory diseases (Cassileth, 2010). Recent research has revealed that *Trifolium pratense L.* possessed more extensive beneficial properties in addition to the above functions, such as preventing the progression of type 2 diabetes and lowering serum lipid and blood pressure (Polak et al., 2015). The products of red clover are also used as dietary supplementation to relieve women’s menopausal symptoms (Andres et al., 2015). *Astragalus membranaceus* (Huangqi) is also a kind of perennial herb, its rhizome has been used as a traditional Chinese medicine for centuries with multiple benefits for health. The main bioactive components of red clover and *Astragalus membranaceus* are flavonoids*,* which can be divided into isoflavones, coumestans and prenylflavonoids from the structure (Krizova et al., 2019). Among the plethora of flavonoids, formononetin has attracted more and more attention because of its antitumor and neuroprotective activities in recent years. Formononetin is a common component of legumes. It is especially rich in red clovers *Trifolium pratense L.*, and *Astragalus membranaceus* (Huangqi). Formononetin, together with daidzein, biochanin and genistein, belongs to isoflavone phytoestrogens, which are a series of non-steroidal phenolic compounds with estrogen-like biological activities (Mu et al., 2009) (Figure 1). Upon oral administration, formononetin can be hydrolyzed along the whole gastrointestinal tract. After being absorbed in the proximal part of the gut through passive diffusion, formononetin is O-demethylated into daidzein quickly, and then daidzein is conversed into β-glucuronides or sulphate esters by the corresponding transferases with conjugation at one or two (4′ or 7′) locations of the isoflavone ring (Ronis et al., 2006). The conjugates (glucuronides and/or sulfates) are the main form of formononetin *in vivo*, indicating by the higher plasma concentration time curve (AUC) comparing to free formononetin (Singh et al., 2011).

*In vivo*, formononetin exerts estrogen-like effects by binding to estrogen receptors (ER) and regulating gene expression of down-stream targets. Like other isoflavones, the binding of formononetin with ER is subtype-selective, the binding affinity to ER *β* is much higher than ERα (Vitale et al., 2013). It has been proved that formononetin is beneficial for attenuating osteoporosis (Ye et al., 2006), cardiovascular diseases (Teede et al., 2001), relieving menopausal symptoms (Lethaby et al., 2007), exerting antitumor and neuroprotective effects (Tay et al., 2019). In view of the shortcomings of chemical drugs for neurological disorders, the good neuroprotective effects of formononetin make it possible to be an alternative medicament for treating these diseases. Thus, an urgent need to understand the neuropharmacological function of formononetin has emerged. Recently, a paper reviewed the signaling pathways under the neuroprotective effects of formononetin, which mainly focused on the molecular targets of formononetin (Geng and Jiang, 2022). In this review, we primarily summarized the biological and pharmacological activities of formononetin in treating neurological disorders, that is, the therapeutic potential of formononetin in preclinical or clinical studies. We also compared the neuroprotective effects of formononetin with other isoflavone phytoestrogens, discussed the pharmacokinetic characteristics and safety profile of formononetin. The synthesis of these scientific evidences about neuroprotective properties of formononetin could facilitate future research to further explore the feasibility of formononetin in clinical therapy.

## Neuroprotective Effects of Formononetin

### Formononetin and Alzheimer’s Disease

Alzheimer’s disease (AD) is a progressive neurodegenerative disorder that accounts for most of the dementia among elderly population. With the coming of aging society, it has been estimated that there will be around 14 million cases of AD by 2060 (Alzheimer’s, 2016). We can foresee that AD will emerge as a major public health issue and bring heavy social burden in the near future. Although the etiology of AD is still obscure, a mounting body of evidence has demonstrated that extracellular deposition of fibrillar β-amyloid (Aβ) peptide and the aggregation of neurofibrillary tangles containing hyper-phosphorylated tau are the main pathological hallmarks of AD (Yankner et al., 1990; Zlokovic, 2005). The therapeutic approaches targeting Aβ peptide or/and neurofibrillary tangles may be effective in controlling the symptoms of AD.

Some studies have investigated the role of formononetin in cellular or animal models of AD. In cultured HT22 hippocampal neuronal cells, formononetin reduced Aβ_25-35_-induced cell apoptosis *via* activating phosphatidylinositol 3-kinase/protein kinase B (PI3K/Akt) pathway. It also increased the activity of α-secretase, which cleaved amyloid precursor protein (APP) into soluble amyloid precursor protein *a* (sAPPα), and then inhibited the accumulation of amyloid plaques (Chen et al., 2017). The effect of formononetin on α-secretase was also demonstrated by using hypoxic human-APP Swedish mutation cell (N2a-APP cell) as an *in vitro* model of AD-like pathology (Sun et al., 2012). Formononetin protected cells against hypoxia-induced neurotoxicity by increasing α-secretase activity and promoting sAPPα secretion *via* upregulating A-Disintegrin-And-Metalloproteinase 10 (ADAM10) expression at the transcriptional level (Sun et al., 2012).

*In vivo*, formononetin was demonstrated to be neuroprotective in the APP/PS1 mice (a mouse model of AD), shown by marked functional improvements in spatial learning and memory in Morris water maze test (Fei et al., 2018). The study of molecular mechanisms revealed that formononetin promoted cerebral Aβ clearance in a low-density lipoprotein receptor-associated protein 1 (LRP1)-dependent pathway and inhibited Aβ accumulation *via* suppressing receptors for advanced glycation-end products (RAGE)/nuclear transcription factor-κB (NF-κB) inflammatory pathway. Another study examined the effects of formononetin on the cognitive function of high-fat diet-induced mice, which mimicked the presymptomatic AD stage (Fu et al., 2019). At the dose of 20 and 40 mg/kg, formononetin significantly attenuated the learning and memory deficits of model mice through inhibiting tau hyperphosphorylation mediated by the activation of glycogen synthase kinase-3β (GSK-3β) pathway in the hippocampus.

### Formononetin and Other Types of Dementia

AD is a special type of dementia. Besides AD, dementia can also be caused by some other factors. Formononetin was also shown to produce neuroprotective effects against dementia induced by specific drugs. Shaza et al found that formononetin markedly increased the discrimination ratio of novel object recognition test in the demented mice induced by scopolamine, and its efficacy was comparable to donepezil. Further research revealed that formononetin inhibited the elevation of dopamine (DA), norepinephrine (NA) and malondialdehyde (MDA) levels, reduction of glutathione (GSH) and acetylcholine (ACh) levels, enhancement of acetylcholinesterase (AChE) activity in the brain of scopolamine-treated mice. Formononetin seemed to play a neuroprotective role in scopolamine-induced dementia through regulating brain AChE activity and suppressing oxidative stress (Aly et al., 2021). Diabetes is a risk factor for the dysfunction of nervous system. The protective effects of formononetin have been examined in a mouse model of diabetic cognitive dysfunction induced by intraperitoneal injection of streptozotocin (STZ). Formononetin attenuated the decline of learning and memory abilities in STZ-treated mice through suppressing inflammatory response mediated by HMGB1/TLR4/NF-κB signaling in the hippocampus (Wang et al., 2018).

Estrogen deficiency is the initial factor of cognitive decline in menopausal women. In a small-sample clinical trial, Howes et al. (2004) assessed the effects of dietary supplementation with isoflavones from red clover on cognitive function in 30 postmenopausal women. Formononetin was the main component of the isoflavones (each tablet containing formononetin 25 mg, biochanin 2.5 mg and less than 1 mg of daidzein and genistein). However, they found that isoflavone supplementation with two tablets a day for 6 months did not appear to have significant effects on cognitive function in postmenopausal women (Howes et al., 2004). The negative results of this trial may be related to the following aspects: First, the six-month testing period may not be too long enough to assess the effect of dietary isoflavones, as all the included women had been postmenopausal for at least 5 years and never received therapy; Second, the dose of isoflavones may not be higher enough to produce sufficient effect as the estrogenic capacity of these phytoestrogens are lower compared to endogenous estrogens. Third, the sample size of this trail was small, some uncontrollable variables may bring deviation to the results and then cover the real effect of the testing substance. Therefore, further larger-sample size and well-designed clinical trials are still needed to clarify the effect of formononetin on cognitive function.

### Formononetin and Cerebral Ischemia

Cerebral ischemia is a devastating neurological dysfunction caused by reduced or cut-off of blood flowing to the brain, and is the second leading cause of death worldwide (Hankey, 2017). The protective effects of formononetin against cerebral ischemia have been examined by multiple *in vitro* and *in vivo* studies. Formononetin protected PC12 cells from oxygen-glucose deprivation/reperfusion (OGD/R) injury by increasing the expression of growth-associated protein 43 (GAP-43) and brain-derived neurotrophic factor (BDNF) (Yan et al., 2019). Formononetin was also shown to protect primary neurons against OGD-induced neurotoxicity by upregulating neuroglobin expression in cAMP response element-binding protein (CREB)-dependent pathway (Liu et al., 2016).

*In vivo*, the neuroprotective effect of formononetin against ischemic stroke was tested in rats subjected to middle cerebral artery occlusion (MCAO). Formononetin promoted the functional recovery of MCAO rats by facilitating the formation of dendritic spines and enhancing synaptic plasticity, which may be mediated by the phosphatidylinositol 3-kinase/protein kinase B/extracellular regulated protein kinases (PI3K/AKT/ERK) signaling pathway (Wu et al., 2020). Another study found that sulphonated formononetin alleviated the brain edema, reduced cerebral infarct volume and improved the neurological function of cerebral ischemia rats by inhibiting cell apoptosis and promoting cerebrovascular angiogenesis (Zhu et al., 2014). Liang et al. (2014) also demonstrated that formononetin protected rats from the cerebral ischemia through inhibiting the function of pro-apoptosis pathway and promoting the function of anti-apoptosis molecules (Liang et al., 2014). Yu et al. (2022) found that formononetin significantly alleviated the neurological deficit and the pathological state of brain tissues in MCAO rats by attenuating inflammatory pathways and reducing the production of pro-inflammatory cytokines (Yu et al., 2022). The above studies suggest that formononetin is a natural compound with the potential to protect brain against ischemic injury.

### Formononetin and Traumatic Brain Injury

Traumatic brain injury (TBI) is a kind of brain damage resulted from the external physical force (Larsson et al., 2013). It is a main cause of brain dysfunction, characterized by structural damage and cell death of the brain, which may result in permanent disability or death (Levy et al., 2005; Durand et al., 2017). The phytomedicine may be effective in managing post-traumatic symptoms.

In rat TBI model, formononetin ameliorated the brain damage by repairing neuronal ultrastructural organization and promoting cortical proliferation in a dose-dependent manner, which was consistent with the functional improvements, such as reduction of injure scores and improvement of neurological symptoms. The levels of interleukin-6 (IL6) and tumor necrosis factor (TNF-α) in the serum were increased, while the levels of interleukin-10 (IL10) in serum and cortical neurons were decreased in the TBI rats. The above changes were reversed following the treatment with formononetin. The neuroprotective effects of formononetin against TBI were achieved by activating IL10-based pathway and suppressing neuroinflammatory reaction in the cortex nearing lesioned tissue (Li et al., 2018). Another study found that pretreatment with formononetin for 5 days remarkably prevented pathological lesions and neuronal apoptosis, attenuated brain edema and improved the neurological scores of TBI rats. Formononetin inhibited the downregulation of heme oxygenase-1 (HO-1) and upregulation of BACH1, which was a core transcriptional repressor protein in the nuclear factor erythroid 2-related factor 2 (Nrf2)/HO-1 signaling pathway. The neuroprotective effects of formononetin was achieved through attenuating the oxidative stress *via* activation of Nrf2-dependent antioxidant pathway (Li et al., 2017).

The anti-infammatory and antioxidant effects of formononetin were also verified by other studies. Li et al. (2014) found that the expression of glutathione peroxidase (GSH-Px), superoxide dismutase (SOD) and Nrf2 were decreased, whereas the concentrations of MDA, TNF-α, IL-6 and expression of cyclooxygenase-2 (COX-2) were elevated in the lesioned brain region of the TBI rats. Formononetin treatment reversed above alterations induced by TBI. Formononetin also attenuated the cellular pathological alterations in the lesioned area of brain and increased the neural cell number, alleviated hydrocephalus and neurological abnormalities in TBI rats. It was confirmed again that formononetin protected the brain against TBI at least partially through elevating the activities of antioxidant cascade and inhibiting the inflammatory response in the injured area (Li et al., 2014).

### Formononetin and Anxiety/Depression

Anxiety and depression are two common mental disorders affecting millions of people globally. The current antidepressant drugs have poor efficacy along with many side effects and cannot meet clinical needs (Dinoff et al., 2017). In the complete Freund’s adjuvant (CFA)-induced mouse model of anxiety, formononetin increased the time spent in the open arms of elevated plus maze test and the central area of open field test compared with the model mice, indicating that formononetin possessed anxiolytic effects. The mechanism study showed that formononetin attenuated inflammation and neuronal hyperexcitability in the basolateral amygdala, which contributed to its anxiolytic effects (Wang et al., 2019). Recently, a study evaluated the antidepressant effect of formononetin on chronic corticosterone-treated mice. The results demonstrated that formononetin alleviated depression-like behaviors of model mice reflected by increasing the sucrose preference index and reducing immobility time in the forced swimming test. Mechanism study revealed that formononetin enhanced the expression level of the glucocorticoid receptor and BDNF, attenuated neuronal impairment and promoted neurogenesis in the hippocampus (Zhang et al., 2022). The above researches laid a foundation for further studying the effects of formononetin on mental illness.

### Formononetin and Neuroinflammation

Although neuroinflammation is not a specific disease, it is the pathological state accompanied by various neurological disorders. As the innate immune cells of the central nervous system, microglia is also the mediator of neuroinflammation. Activated microglia promoted the production of TNF-α, IL-6, IL-1β, which are pro-inflammatory cytokines.

The effects of formononetin on neuroinflammation were investigated in lipopolysaccharide (LPS)-stimulated BV2 mouse microglia cell line. Formononetin was found to reduce the level of TNF-α, IL-6, IL-1β significantly and also inhibit COX-2-mediated prostaglandin E2 (PGE2) production and inducible nitric oxide synthase (iNOS)-mediated nitrite production. Mechanisms research revealed that formononetin exerted the anti-neuroinflammation effects through inhibiting NF-κB signalling pathway. Formononetin also showed protective effects in HT22 hippocampal neurons against the neurotoxicity induced by microglia conditioned medium, which may be through the mechanisms involving estrogen receptor *β* (El-Bakoush and Olajide, 2018). In rat mesencephalic neuron-glia co-cultures, formononetin prevented reduction of dopamine uptake and loss of dopaminergic neurons *via* suppressing microglia activation and production of pro-inflammatory and pro-oxidant factors (Chen et al., 2008).

*In vivo*, formononetin significantly inhibited the production of TNF-α and IL-1β in the hippocampus of high-fat diet-induced mice in a dose-dependent manner. For the mechanisms underlying it, Fu *et al* revealed that formononetin activated anti-inflammatory Nrf-2/HO-1 signaling pathway, and inhibited the pro-inflammatory NF-κB signaling pathway (Fu et al., 2019). The above results suggested that the anti-neuroinflammatory effect of formononetin is one important mechanism underlying its neuroprotective property.

### Formononetin and Pathological Nociception

Peripheral neuropathy is a common comorbidity of type 2 diabetes mellitus (T2DM), with more than half of diabetic patients developing neuropathy within few years after diagnosis (Khawaja et al., 2018). It is an important reason for the decline of life quality and amputation of foot or toe. In the rat model of T2DM induced by high-fat diet and streptozotocin administration, formononetin improved the nerve conduction, inhibited thermal hyperalgesia and mechanical allodynia markedly. Mechanism research found that formononetin may ameliorate hyperglycemia-induced neuropathic condition in T2DM rats by activating sirtuin1(SIRT1) and nerve growth factor (NGF) pathway in sciatic nerve tissue (Oza and Kulkarni, 2020). Another study found that formononetin alleviated oxaliplatin-induced peripheral neuropathy by inhibiting nociceptive sensations to cold and mechanical stimuli. Mechanism study revealed that formononetin prevented oxaliplatin-induced neuronal apoptosis *via* activating Nrf2/glutathione S-transferase pi 1 (GSTP1) signaling pathway (Fang et al., 2020).

Formononetin markedly reduced the number of abdominal writhes and produced evident antinociceptive effects in acetic acid writhing test. In the glutamate-induced nociceptive model, formononetin produced analgesic effect by inhibiting the licking response in the glutamate-injected paw of mice. Formononetin also suppressed oedema response and leukocyte migration caused by carrageenan, and played a significant anti-inflammatory role (Lima Cavendish et al., 2015). However, in the complete Freund’s adjuvant (CFA)-induced chronic inflammatory pain model, formononetin administration remarkably alleviated pain-related anxiety-like behaviors but did not inhibit the mechanical allodynia and thermal hyperalgesia of model mice (Wang et al., 2019). The discrepancy may be due to the different methods in inducing pain models and/or the dosage/duration of formononetin administration.

The pharmacological activities of formononetin against nervous system related disorders are shown in Table 1.

**TABLE 1:** Pharmacological activities of formononetin in nervous system related disorders.

Disease model	Study sample	Concentration or dose	Result	References
Alzheimer’s Disease	Aβ_25-35_-treated HT22 cells	5 μM	↑α-secretase activity,**↑**sAPPα,**↑**cell viability	Chen et al. (2017)
			↓APP,**↓**amyloid plaques,**↓**cell apoptosis	
	Hypoxic N2a-APP cell	10 μM	**↑**Activity of α-secretase,**↑**sAPPα	Sun et al. (2012)
			**↑**ADAM10 protein level,**↓**Cell apoptosis	
	APP/PS1 mice	15 mg/kg/d for 30 days	**↓**Aβ production, **↑**Cerebral Aβ clearance	Fei et al. (2018)
			**•** Formononetin suppressed neuroinflammation	
			**↑**Learning and memory ability	
	high-fat diet mice (presymptomatic AD stage)	20 and 40 mg/kg/d for 10 weeks	**↓**Blood glucose, total cholesterol and triglyceride;**↓**Levels of IL-1β and TNF-α	Fu et al. (2019)
			**•** Formononetin suppressed pro-inflammatory signaling pathways	
			**↓**Tau hyperphosphorylation	
			**•** Formononetin significantly attenuated the learning and memory deficits	
Other types of Dementia	Scopolamine-treated mice	Pretreatment with 50 mg/kg	**↓**Levels of DA,NA;**↓**AChE activity	Aly et al. (2021)
			**↓**MDA level, **↑**GSH level	
			**↑**Novel object recognition discrimination ratio	
	STZ-treated mice	25 and 50 mg/kg/d for 6 weeks	**↓**Body weight; **↓**Blood glucose	Wang et al. (2018)
			**•** Formononetin suppressed inflammatory pathway	
			**↓**Levels of IL-1β, IL-6,TNF-α,MDA	
			**↑**Learning and memory ability	
Cerebral ischemia	PC12 cells with OGD/R injury	0.163, 1.630, 16.300 μg/ml	**↑**GAP43 and BDNF expression	Yan et al. (2019)
			**↑**Cell viability	
	OGD-treated primary neurons		**↑**Neuroglobin and CREB expression	Liu et al. (2016)
			**↑**Cell viability	
	MCAO rats	30 mg/kg for 2 weeks	**↑**Levels of GAP43, BDNF,NGF	Wu et al. (2020)
			**↑**Number of neuronal dendritic spines	
			**•** Formononetin improved the long-term functional recovery	
	MCAO rats	3, 7.5, 15, and 30 mg/kg	**↓**Brain water content; **↓**Infract volume	Zhu et al. (2014)
			**↓**Cerebral cell apoptosis	
			**↓**Neurological deficit scores	
	Rats with focal cerebral I/R	12.5, 25, 50 mg/kg/d for 2 weeks	**↓**Brain water content;**↓**Infract volume	Liang et al. (2014)
			**↓**Neurological deficit scores	
			**↑**Levels of ERα, Bcl-2,p-AKt,**↓**Bax	
TBI	TBI model rats	10,30 mg/kg/d for 7 days	**•** Formononetin improved neurological symptoms of TBI rats	Li et al. (2018)
			**↑**Neuronal proliferation	
			**•** Formononetin improved neuronal ultrastructural organization	
			Levels of IL-10 in serum and cortex	
	TBI model rats	10,30 mg/kg/d for 5 days	**•** Formononetin promoted functional recovery	Li et al. (2017)
			**↓**the edema and necrosis;**↓**neuronal apoptosis	
			**↑**HO-1 level,**↓**BACH1 level	
	TBI model rats	10, 20 mg/kg/d for 5 days	**•** Formononetin improved neurological symptoms of TBI rats	Li et al. (2014)
			**↓**Cerebral moisture content	
			**↓**The edema and necrosis; ↑Neuronal number	
			**↑** GSH-Px and SOD activities	
			**↓**MDA, TNF-α and IL-6 level	
			**↓**Cox-2 level,**↑** Nrf2 level	
Anxiety/Depression	CFA-injected mice	25 mg/kg/d for 10 days	**↑**Time spent in open arms of elevated plus maze	Wang et al. (2019)
			**↑**Time spent in central area of open field	
			**↓**Expression level of glutamate receptors in basolateral amygdala	
			**↓**Expression level of p65 and Iba-1 in basolateral amygdala	
	corticosterone-injected mice	20, 40 mg/kg/d for 3 weeks	**↑**Sucrose preference;**↓**The immobility time in the forced swimming test	(Zhang et al., 2022)
			**↓**Serum corticosterone level	
			**↑**Expression level of glucocorticoid receptor and BDNF in hippocampus	
			**↓**Neuronal damage	
			**↑**Neurogenesis in hippocampus	
Neuroinflammation	LPS-stimulated BV2 cells	2.5, 5 and 10 μM	**↓**Levels of IL-1β、IL-6、 TNF-α	El-Bakoush and Olajide, (2018)
			**↓**iNOS、Cox-2 and PGE2 level	
			**↑** ERβ level	
	LPS-stimulated neuron-glia co-cultures	0.25, 1.0, and 2.5 μM	**↑**Dopamine neuron number	Chen et al. (2008)
			**•** Formononetin suppressed microglia activation	
			**↓**Levels of TNF-α、NO and superoxide	
	high-fat diet mice	20, 40 mg/kg/d for 10 weeks	**•** Formononetin activated anti-inflammatory Nrf-2/HO-1 pathway, and inhibited the pro-inflammatory NF-κB signaling pathway	Fu et al. (2019)
			**↓**Levels of IL-1β and TNF-α	
Nociception	type 2 diabetic rats	10, 20, and 40 mg/kg/day for 16 weeks	**↓**Thermal hyperalgesia and mechanical allodynia;**↑**Nerve conduction velocity	Oza and Kulkarni, (2020)
			**↑**GSH and SOD level, ↓MDA level	
			**↓**Axonal degeneration, axonal swelling and lymphocytic infiltration	
	oxaliplatin-induced peripheral neuropathy	10 mg/kg	**↓**Cold hyperalgesia and mechanical allodynia	Fang et al. (2020)
			**↓**DRG neuron nucleolar area shrinkage, obvious cytoplasmic vacuolization, and Nissl stain fragmentation	
	nociceptive and inflammatory (carrageenan-induced hindpaw oedema and peritonitis) mouse models	10 mg/kg	**↓**Number of abdominal writhes	Lima Cavendish et al. (2015)
			**•** Formononetin inhibited the nociception induced by glutamate	
			**•** Formononetin inhibited the second phase of formalin-induced nociception	
			**•** Formononetin inhibited oedema response	
			**•** Formononetin inhibited carrageenan-induced leukocyte migration	
	CFA-induced inflammatory pain mouse	25 mg/kg/d for 10 days	**•** Formononetin did not affect mechanical allodynia and thermal hyperalgesia	Wang et al. (2019)
			**•** Formononetin inhibited NF-κB signaling pathway in the BLA.	

## Formononetin *Versus* Other Isoflavone Phytoestrogens

Apart from formononetin, other multiple isoflavones are also commonly used as health supplementation for their beneficial properties (Messina, 2010; Zhang et al., 2019). These isoflavones can bind to estrogen receptors (ER) and display estrogen-like effects although their binding affinities are relatively lower than 17β-estradiol (Cederroth and Nef, 2009). Several studies compared the neuroprotective effects of formononetin with other isoflavone phytoestrogens. The protective effects of formononetin and other four isoflavones (daidzein, calycosin, pratensein and irilone) were examined with the model of LPS-induced dopaminergic neurodegeneration (Chen et al., 2008). All five isoflavones promoted the survival of dopaminergic neurons, suppressed LPS-induced microglia activation and lowered the production of TNF-α, nitric oxide and superoxide. As for the neuroprotective potency, it decreased gradually in the order of pratensein, daidzein, calycosin, formononetin, irilone at the same concentration (Chen et al., 2008). Yu et al. (2005) investigated the neuroprotective effects of formononetin, ononin and calycosin using PC12 cells, and found that they attenuated glutamate-induced cell death, with an estimated 50% effective concentration (EC50) of 0.027 μg/ml, 0.047 μg/ml and 0.031 μg/ml, respectively (Yu et al., 2005). In addition, both formononetin and calycosin significantly increased the activity of SOD and GSH-Px as well as the level of GSH. Formononetin had a greater effect on GSH-Px and GSH, while calycosin had a greater effect on SOD (Yu et al., 2009). Recently, another study investigated the beneficial effects of formononetin, daidzein (the metabolite of formononetin) and calycosin (another major isoflavone from *Astragali Radix*) in cerebral ischemia-reperfusion injury (Gu et al., 2021). The results showed that they protected the primary cultured neurons against oxygen-glucose deprivation plus reoxygenation (OGD/RO) or L-glutamate treatment with an efficacy order: daidzein > formononetin > calycosin (Gu et al., 2021). Their neuroprotective effects were also evaluated by *in vivo* middle cerebral artery occlusion (MCAO) model. It was found that daidzein, formononetin, calycosin at higher dose (60 μmol/kg) reduced the infarct volumes significantly to 13.84 ± 0.95%, 10.76 ± 4.21% and 10.99 ± 6.07%, respectively, compared with vehicle treatment group (27.93% ± 7.1%) (Gu et al., 2021). However, the overall pharmacological outcome of these isoflavones in the “real world” application may be affected by multiple factors, such as the concentration/dose, physical condition of the user, age, sex, food, and so on. Therefore, the neuroprotective efficacy of these isoflavone phytoestrogens are not set in stone and may be varying under different conditions.

## The Pharmacokinetics, Metabolism, and Bioavailability of Formononetin

The lipophilicity is an important factor that determines the absorption, distribution, accumulation and elimination of drugs *in vivo*. The logarithm of octanol/water partition coefficient is commonly used as the physico-chemical parameter to characterize lipophilicity, which is usually determined by the true partition coefficient (log P) value and the distribution coefficient (log D^pH^). Formononetin shows favorable log P and log D^7.4^ value regarding drug-likeness and Lipinski’s rule of five, suggesting that it can passively permeate through biological membranes easily (Gampe et al., 2022). In the central nervous system, lipophilicity affects the amount of drugs passing through the blood-brain barrier (BBB). As for evaluating the BBB permeability, the results of parallel artificial membrane permeability assay (PAMPA) shows that formononetin has a good permeation capability (Gampe et al., 2022), which is the foundation of exerting protective effects against neurological disorders.

Upon oral administration, formononetin is rapidly absorbed and it shows a better permeability in the large intestine segments than small intestine segments. However, the small intestine is still the main site for absorbing formononetin since it is much longer than large intestine, resulting in a larger absorption area and longer residence period (Luo et al., 2018). The plasma concentration of formononetin reaches peak 30–60 min after oral administration, its plasma protein binding rate is about 95% at the physiological concentration (Singh et al., 2011). The maximum plasma concentration (C_max_) of formononetin ranged from 62 to 302 nM after being taken orally at the dose of 20–50 mg/kg, while it could reach nearly 1,303 nM and 16,957 nM after being administered intravenously at the dose of 4 and 10 mg/kg, respectively (Singh et al., 2011; Luo et al., 2018). The apparent volume of distribution (Vd) of formononetin is 14.16 L/kg in rats, which is significantly higher than the total volume of bodily fluids, suggesting an extensive tissue distribution of formononetin (Singh et al., 2011). The liver is the main place of formononetin metabolism, it is demethylated to daidzein by cytochrome P450, and both formononetin and its metabolite daidzein then subjected to phase II conjugation reactions (glucuronidation and/or sulfation) (Chen et al., 2005; Singh et al., 2011), which are the main existing form in plasma after oral and intravenous administration. Formononetin is eliminated with a half-life of nearly 2 h (Luo et al., 2018), and found to have a high systemic total body clearance (CL_Total_) (5.13 L/h/kg in rats), indicating that formononetin is a high extraction compound. Formononetin has a moderate bioavailability of 21.8% upon oral administration at 20 mg/kg, which is higher than many other flavonoids and isoflavones (Luo et al., 2018). It is mainly due to the structure of methylated isoflavone, which confers formononetin a slower glucuronidation and higher gut permeability compared with other unmethylated ones, resulting in a higher bioavailability and tissue distribution (Jia et al., 2004; Walle et al., 2007).

The bioavailability of formononetin may be lower compared with many other chemical drugs for treating neurological disorders. However, long-term dietary supplementation may still raise significant concentrations in plasma and nervous system, and then produce corresponding pharmacological effects (D'Archivio et al., 2010; Di Lorenzo et al., 2021). Moreover, the metabolites of formononetin also have certain biological activities and may become an important source of cellular aglycones upon enzymatic hydrolysis at the target site (Zhang et al., 2007; Barnes et al., 2011). Then the synergistic effects between formononetin and its metabolites may further strengthen the pharmacological functions and endow it good neuroprotective properties.

## Safety and Toxicity

The acute toxicity of formononetin has been investigated by intravenous administration of sodium formononetin-3-sulphonate (Sul-F), a water-soluble derivate of formononetin, in rats and dogs. Sul-F at the dosage of 2000 mg/kg showed no significant body weight change, apparent toxic effects or mortality in rats during the following 2 weeks period. However, non-metabolic Sul-F was found after urine volatilization at the first day after Sul-F administration (Li et al., 2015). In the dogs, 1000 mg/kg Sul-F had no effect on the results of electrocardiogram examinations, ophthalmic examinations, and body weight. However, the transient vomiting was observed at 15–20 min after administration of Sul-F. Pathological examination showed that Sul-F had no evident effects on the main organs such as brain, heart, liver, kidney, stomach, spleen, and intestines. As for the biochemical index, the decrease of phosphocreatine kinase (CK) and the increase of total protein (TP) level were observed in Sul-F treated dogs at the end of the experiment (Li et al., 2015).

The Subchronic toxicity of formononetin has also been assessed using its water-soluble derivate, Sul-F. Dogs were administrated with Sul-F at dose of 0, 33.3, 100, and 300 mg/kg/day for 90 days followed by a four-week recovery period. No mortality, ophthalmic abnormalities, changes of body weight, evident alterations of biochemical and histopathological parameters were observed except that transient vomiting and recoverable vascular stimulation were detected in the group of 300 mg/kg/day (Li et al., 2016).

Although there is still no study reporting the LD50 of formononetin, it is not difficult to get the conclusion that formononetin has a good safety profile from the above acute and subchronic toxicity studies, because the dose above 300 mg/kg is very high and it only elicit transient and mild adverse effects, with no apparent toxicity being reported. Even so, further detailed and more rigorously designed studies are still needed to uncover other potential toxic and side effects of formononetin.

## Conclusion and Future Perspectives

Increasing evidences have suggested that phytoestrogens exert neuroprotective effects in neurological disorders. As an important phytoestrogen and also the main bioactive component of dietary foods such as soybean, alfalfa beans and chickpeas, formononetin may be a suitable candidate for the treatment of neurological disorders with a good safety profile.

**FIGURE 2:**
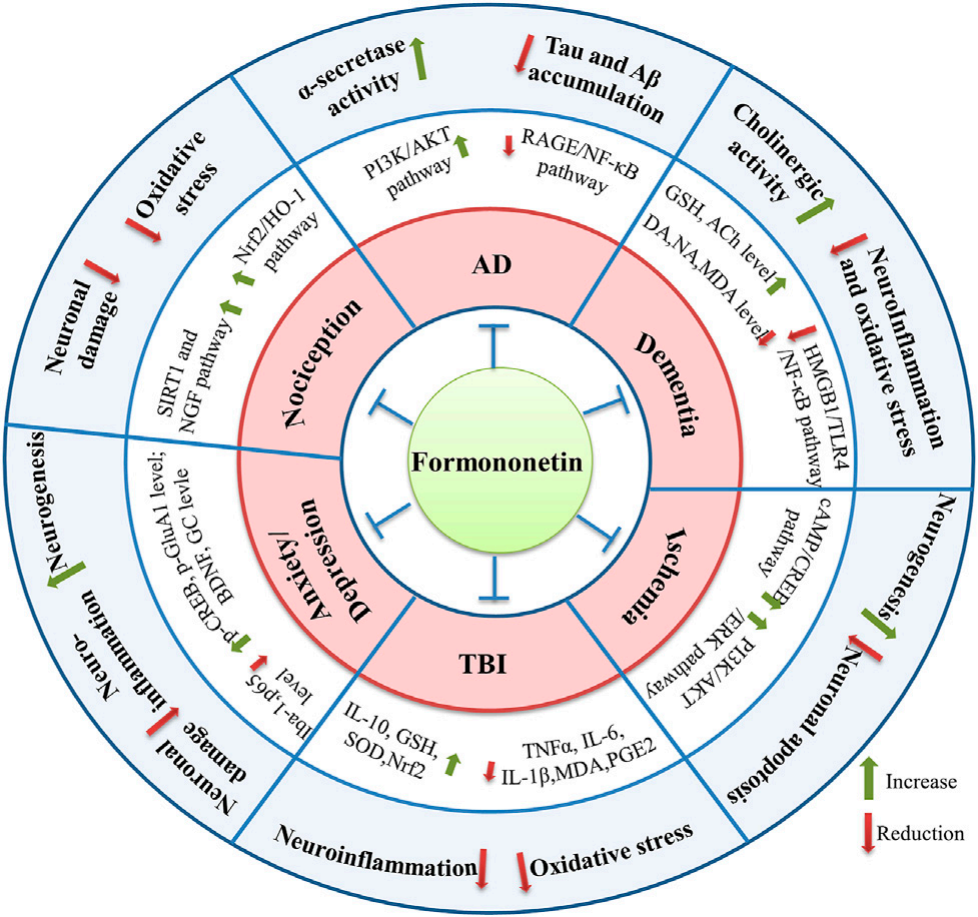
The molecular targets involved in the neuroprotective effects of formononetin.

**FIGURE 3:**
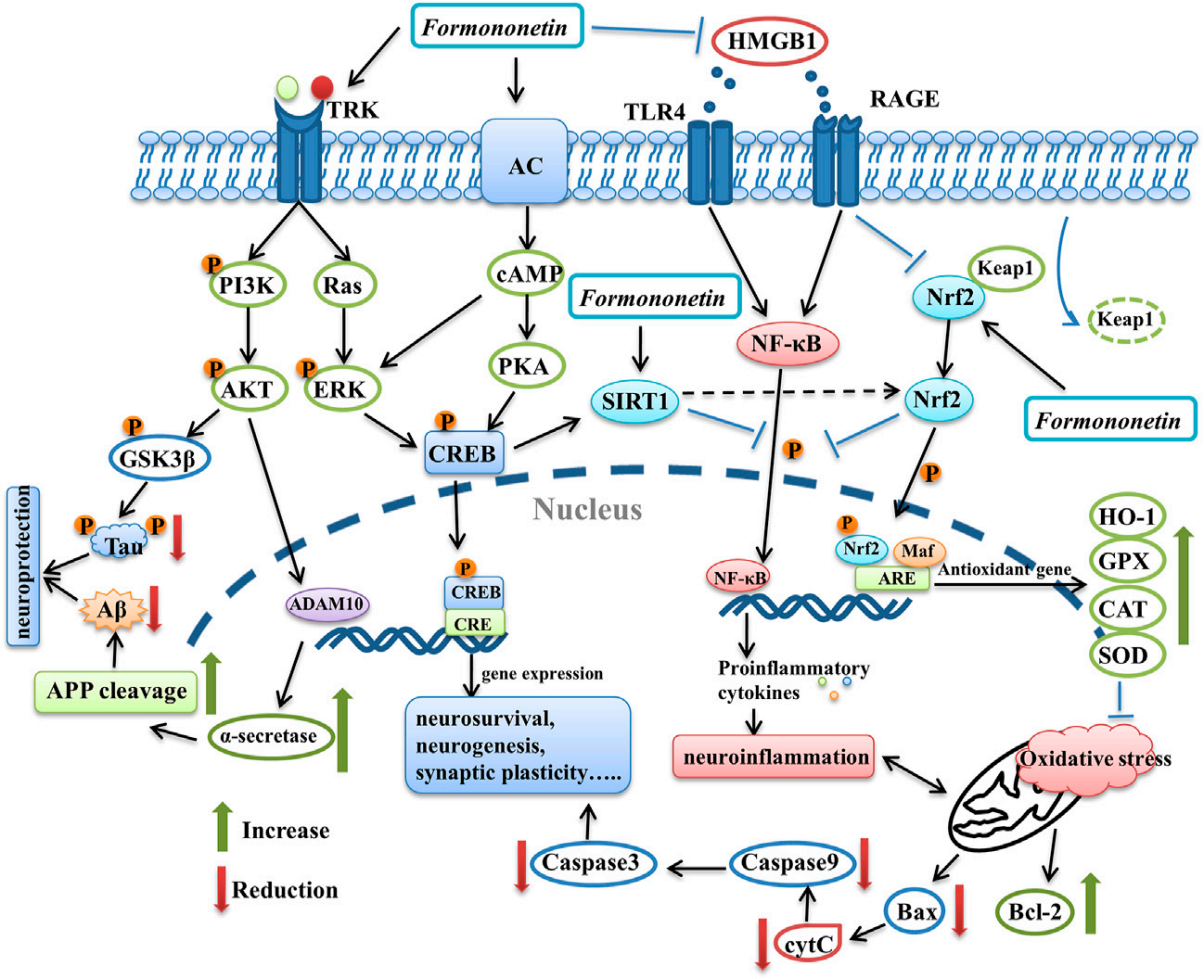
The graphical summary of signaling pathways underlying the neuroprotective effects of formononetin. It benefits multiple aspects of nervous system, such as promoting neurosurvival, neurogenesis and synaptic function *via* modulation PI3K/AKT pathway, ERK pathway, PKA/CREB pathway. Formononetin attenuates neuroinflammation through inhibiting HMGB1/TLR4/NF-κB pathway and RAGE pathway. It can also inhibit oxidative stress and neuronal apoptosis through activating Nrf2 pathway. In addition, SIRT1 is also involved in the neuroprotective effects of formononetin. PI3K, phosphatidylinositol 3-kinase; AKT, protein kinase B; GSK3β, glycogen synthase kinase-3β; ADAM10, A-Disintegrin-And -Metalloproteinase 10; APP, amyloid precursor protein; AC, adenylate cyclase; cAMP, cyclic adenosine monophosphate; PKA, protein kinase A; CREB, cAMP response element-binding protein; TRK, receptor tyrosine kinase; HMGB1, high mobility group box-1 protein; TLR4, toll-like receptor 4; RAGE, receptors for advanced glycation-end products; NF-κB, nuclear factor kappa-B; Nrf2, nuclear factor erythroid 2-related factor 2; HO-1, heme oxygenase-1; GPX, glutathione peroxidase; CAT, catalase; SOD, superoxide dismutase.

In this review, the beneficial neuropharmacological effects of formononetin in both *in vitro* and *in vivo* studies were summarized. Formononetin showed promising effects in a series of neurological disorders such as AD, pathological pain, TBI, anxiety/depression, cerebral ischemia, dementia, and so on. The protective efficacy of formononetin was compared with some other isoflavone phytoestrogen, the pharmacokinetic characteristics and safety profile of formononetin were also discussed in this paper. Numerous molecular targets and pharmacological mechanisms are involved in the neuroprotective property of formononetin. The potential molecular targets and signaling pathways underlying neuroprotective effects of formononetin are briefly summarized in Figure 2 and Figure 3, respectively. Actually, some signaling pathways modulated by formononetin had been summarized in a recent paper, which mainly focused on the biological mechanisms of formononetin at the molecular level (Geng and Jiang, 2022). Our review primarily discussed the pharmacological effects and important pharmacological parameters of formononetin based on diseases, not just only signaling pathways. They complement each other and will facilitate future studies to further investigate the neuroprotective properties of formononetin.

Although multiple preclinical studies have demonstrated independently that formononetin possessed therapeutic potentials against a wide range of neurological disorders, it also has many problems that remain to be solved. First, formononetin is absorbed rapidly and undergoes extensive metabolism upon administration, the metabolites may have varying bioactivities and pharmacokinetic properties, which will affect the overall pharmacological outcomes. It is essential to determine the amount of formononetin and its bioactive metabolites reaching the target tissues. However, few studies have attempted to investigate formononetin and its metabolites qualitatively and quantitatively *in vivo*. Therefore, future studies should focus more on clarifying the pharmacokinetic characteristics of the parent molecule and its metabolites, and evaluating their influence on the overall bioactivity of formononetin. Second, the safety profile of formononetin should be further confirmed. Although the acute and subchronic toxicity of formononetin have been assessed, the data comes from the same laboratory, it would be more convincing if there were data from multiple different laboratories. On the other hand, there is no data on the long-term safety of formononetin, which needs to be fully elucidated to ensure that this bioactive isoflavone is safe for clinical development. Third, the lack of clinical trials on the therapeutic effects of formononetin is another important limitation. So, detailed and well-designed clinical studies are needed in future research to assess the efficacy and safety of formononetin in treating neurological disorders. This is crucial because the pharmacodynamic and pharmacokinetic properties of formononetin may be diverse between animals and humans, as well as the different physiological states of the body.

Taken together, the promising neuropharmacological effects of formononetin strongly indicate that it may be a potential candidate for treating neurological diseases, though some clinical aspects, such as bioavailability, appropriate dose, tolerability, and efficacy should be further clarified before expanding formononetin treatment into humans.
